# Supplementary material for: Nitrate restricts nodule organogenesis through inhibition of cytokinin biosynthesis in Lotus japonicus
Source: Nat Commun. 2021 Nov 11;12:6544. doi: 10.1038/s41467-021-26820-9 (PMC8585978; doi:10.1038/s41467-021-26820-9)
Supplement: Supplementary file 1 — Supplementary Information [file 41467_2021_26820_MOESM1_ESM.pdf]

Supplementary figures and tables

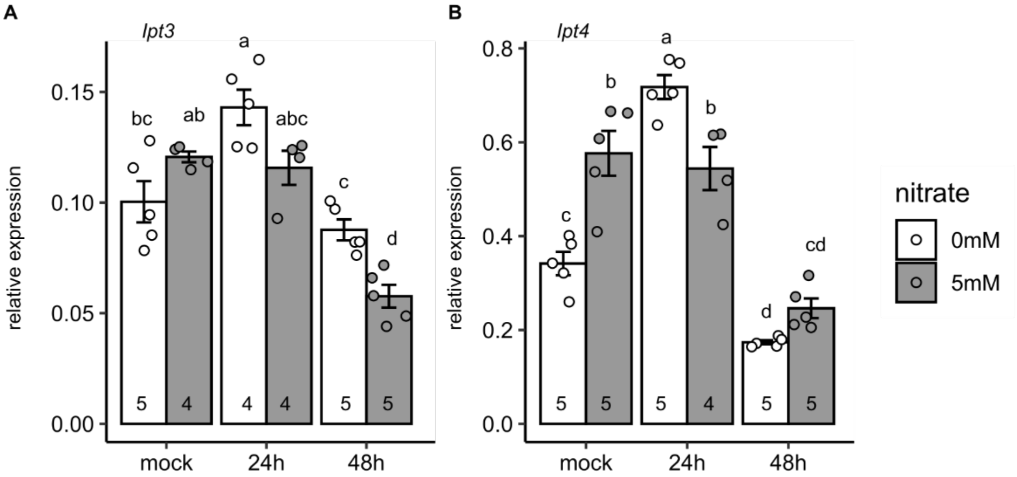

Supplementary Fig 1. Nitrate inhibits induction of symbiotic cytokinin biosynthesis. Relative transcript abundance by qRT-PCR 1 and 2 days post inoculation (dpi) with *M. loti* in absence and presence of 5 mM KNO_3. Bars show mean +/-SE for *n* values as shown below the respective groups in qRT-PCR. Significant differences between nitrate presence and absence conditions is indicated by different letters (p<0.05) as determined by ANOVA and Tukey post-hoc testing with pairwise P-values available in the Source Data file.

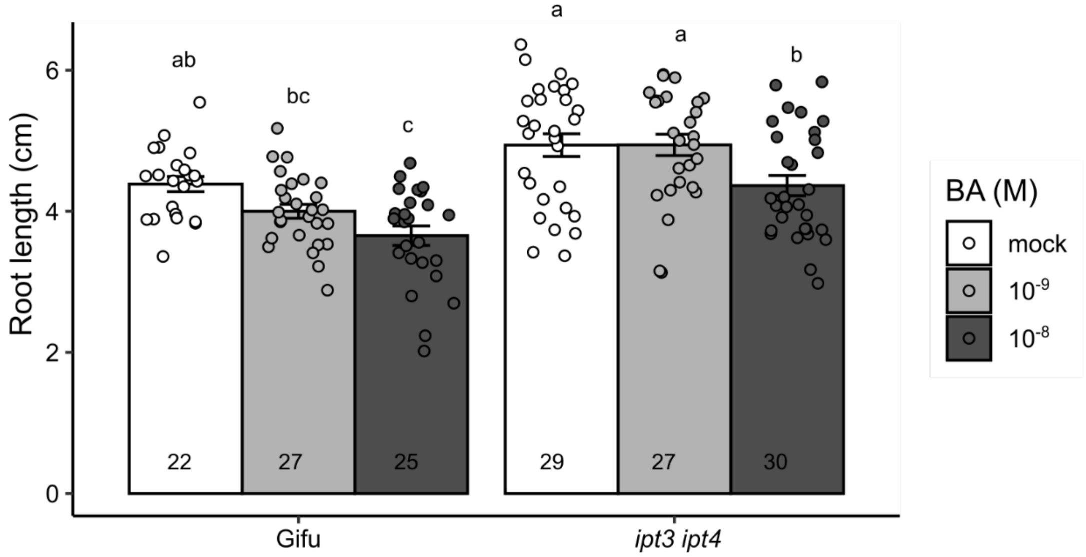

Supplementary Fig 2. *ipt3 ipt4* retains sensitivity to cytokinin inhibition of root length. Primary root length in the presence of 5 mM KNO_3 with mock, 10^{-9} or 10^{-8} M BA at 7 days after germination of the indicated host genotypes. *n* is indicated on the bars. Significant differences among different genotypes and concentration of BA are indicated by letters (p<0.05) as determined by ANOVA and Tukey post-hoc testing with pairwise P-values available in the Source Data file.

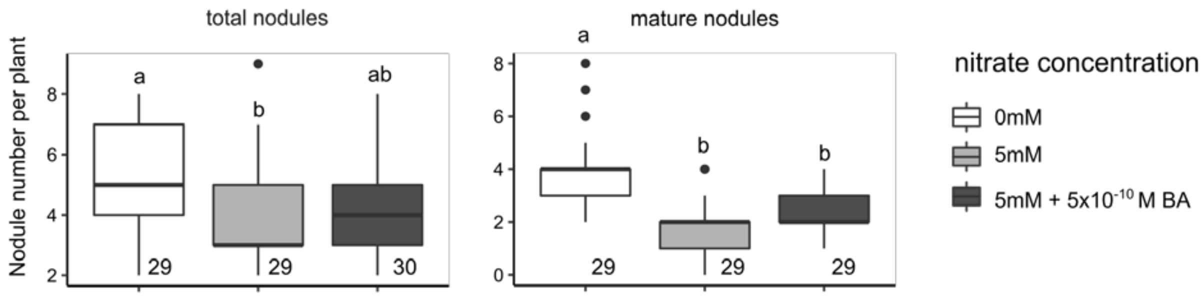

Supplementary Fig 3. Nodule numbers in the presence of 5 mM KNO_3 with $5x10^{-10}$ M BA at 14 days after inoculation with *M. loti*. Significant differences among different treatments are indicated by letters ($p<0.05$) as determined by ANOVA and Tukey post-hoc testing for *n* as indicated below boxplot. Box plots show Min, Q1, Median, Q3, Max and outlier values. Pairwise P-values are available in the Source Data file

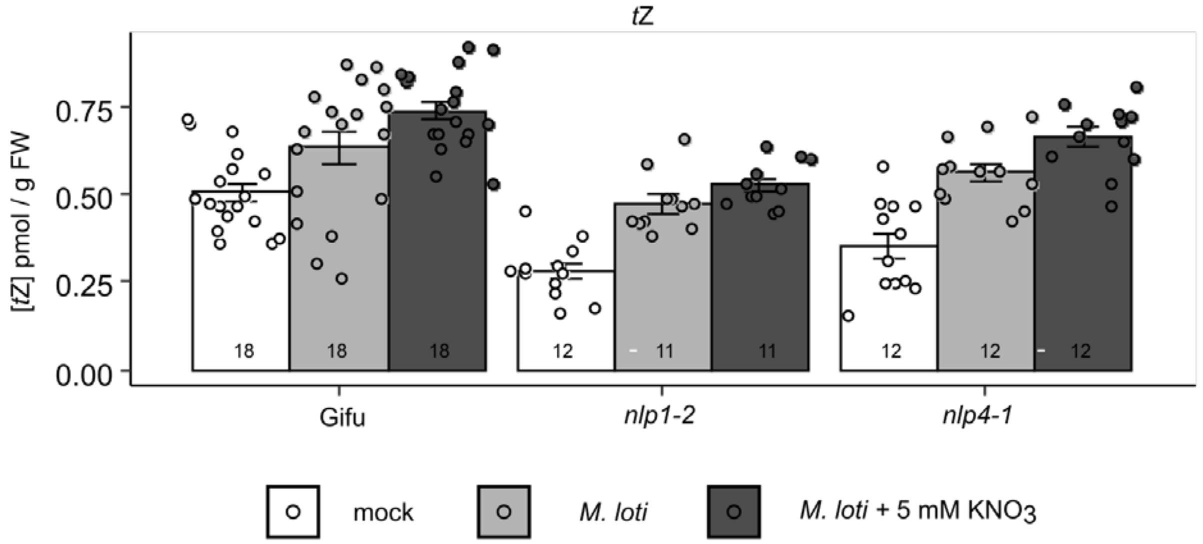

Supplementary Fig 4. Cytokinin *t*Z free base content in the indicated genotypes at 2 dpi with *M. loti*. Bars show mean +/-SE for *n* indicated on the bars . No significant differences were found between nitrate presence and absence conditions as determined by Wilcoxon rank-sum test.

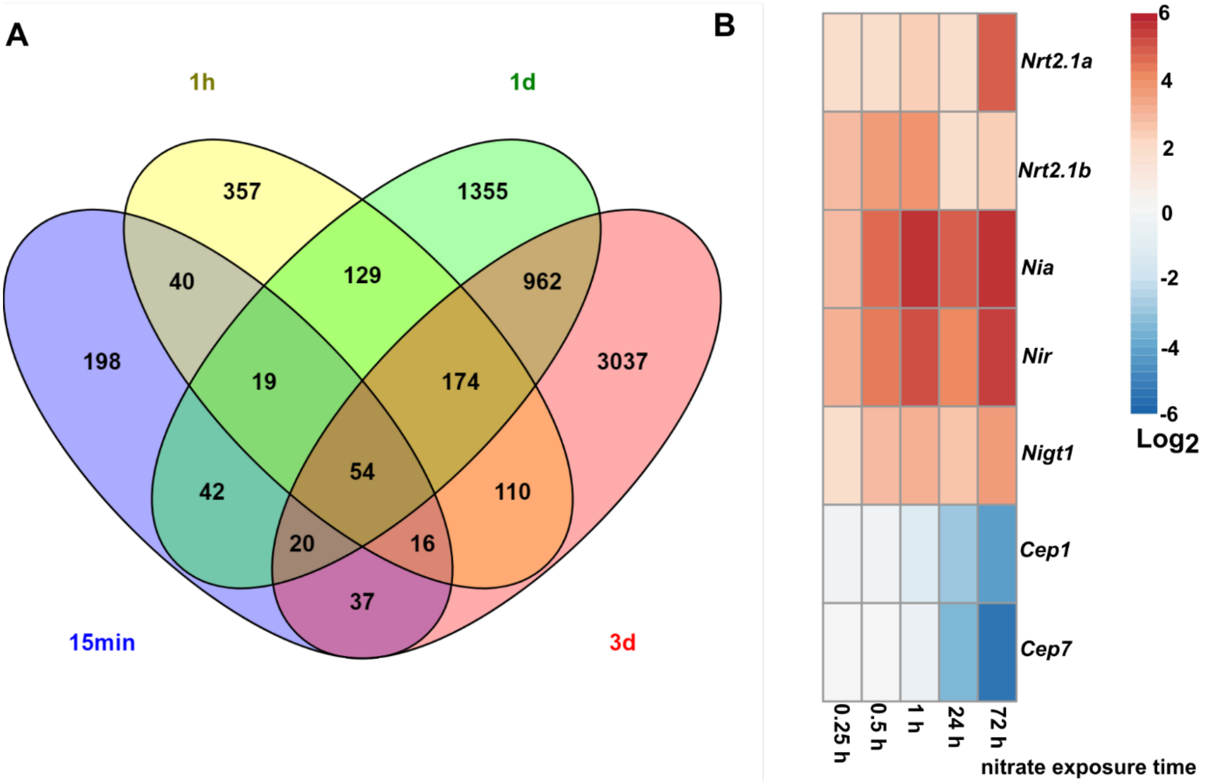

Supplementary Fig 5. Nitrate response in RNAseq study. A, A Venny diagram [67] of different expression genes under time series of nitrate exposure. Differentially expressed genes are those with an adjusted $p<0.1$. B, Heatmap of nitrate marker gene expression under the time series of nitrate exposure. The expressions are normalized to mock treatment.

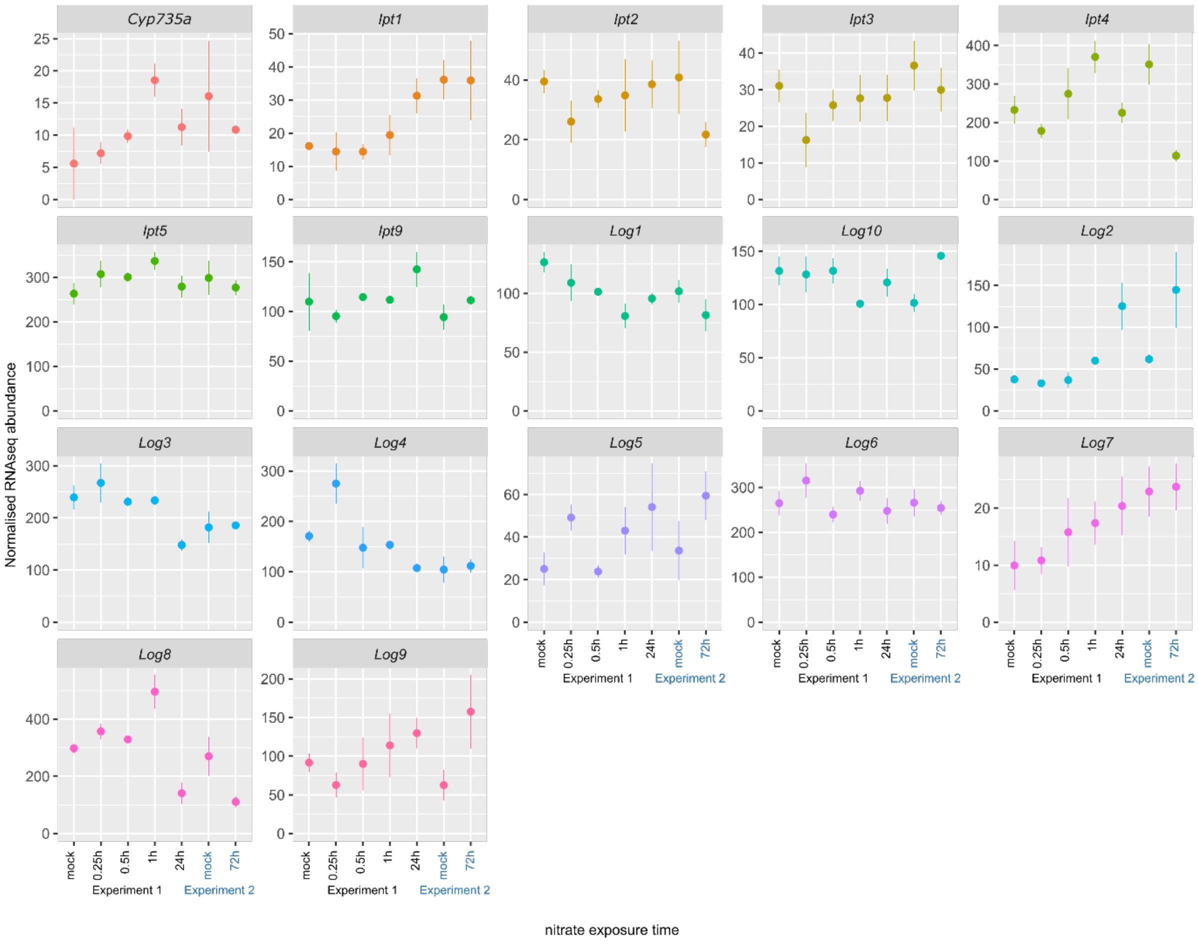

Supplementary Fig 6. Cytokinin biosynthesis gene responses to nitrate exposure. Transcript abundance determined by RNAseq of Cyp735, *Ipt* and*Log* genes following nitrate exposure at the indicated times. Values are mean normalised transcript abundance +/- SE for n=3 replicates.

Supplementary table 1: Gene ID and primers used in this study

Gene name	ID	qPCR primer	qPCR primer
Ubi	LotjaGi1g1v0401300	atgtgcattttaagacaggg	gaacgtagaagattgcctgaa
Ipt2	LotjaGi4g1v0351300	GAGGAAGCAGAGAAGGTTTGG	CTGATGGGGTACTTGAATTCG
Ipt3	LotjaGi5g1v0164300	GGAAGGTGGTGGCAGAGCC	CATAACACTCTCCGTTGAAC
Ipt4	LotjaGi6g1v0298900	GACTCTGCTGATTCGGAGG	CCACCTGCGGTGACCTA
Log1	LotjaGi1g1v0735700	TATATCCCCCACAGCTCGTC	CTGTCTTCCCACACCAATTT
Log4	LotjaGi1g1v0427900	AGCTTGAGGAATATGTCC	CTGCTATCGGTGTAAATGAA
Nfr1	LotjaGi1g1v0062600	cccttgtaccacagaacc	gctttctcttcttccttcttctg
Nfr5	LotjaGi2g1v0394950	TCATATGATGGAGGAGTTGTCTGTT	ATATGAGCTTCGGAGCATGG
Nsp2	LotjaGi1g1v0257100	ATATGAGCTTCGGAGCATGG	GAGATCTGAAGCGATTTAACAGC
Ern1	LotjaGi1g1v0643700	CCACCCTTGTGCTCATTGTTCTG	CCTACACTCCTCCCTCTCAAG
RR5	LotjaGi4g1v0236600	TCTTGACTCGAATTGATAGGTGC	GATAGAGATGGCCTGCAACTACTG
RR9	LotjaGi1g1v0666300	ATCAGGAAGTGGAGGTGAATC	TCCTTCCTCCAAGCATCTGT
Nin	LotjaGi1g1v0001500	aggagcccaagtgagtgcta	gccatcaaggtatatgacgag
Cyp735a	LotjaGi1g1v0550400	tctccaaactcactctattgc	cagcacaggaatccaaattg
Symrk	LotjaGi2g1v0330500		
Hmgr	LotjaGi2g1v0352700		
Nup85	LotjaGi1g1v0175100		
Nup133	LotjaGi1g1v0009600		
Castor	LotjaGi1g1v0800800		
Pollux	LotjaGi6g1v0363700		
Ccamk	LotjaGi3g1v0307700		
Cyclops	LotjaGi2g1v0343300		
Nsp1	LotjaGi3g1v0414350		
Nrt2.1a	LotjaGi3g1v0487600		
Nrt2.1b	LotjaGi3g1v0487700_LC		
Nia	LotjaGi1g1v0152000		
Nir	LotjaGi4g1v0074400		
Nigt1	LotjaGi2g1v0280600		
Cep1	LotjaGi4g1v0106500		
Cep7	LotjaGi4g1v0241000		
IPT1	LotjaGi5g1v0339400		
IPT5	LotjaGi3g1v0480000		
IPT9	LotjaGi3g1v0156900		
LOG2	LotjaGi1g1v0690800		
LOG3	LotjaGi5g1v0102900		
LOG5	LotjaGi3g1v0164600		
LOG6	LotjaGi1g1v0368200		
LOG7	LotjaGi2g1v0358500		
LOG8	LotjaGi3g1v0551100		
LOG9	LotjaGi5g1v0079100		
LOG10	LotjaGi6g1v0150400		

Supplementary table 2: mutants used in this stuc

mutant	mutant ID	Fwd genotyping primer	Rev genotyping primer
ipt3-1	1893	ACCCTTCCCGCCGGAAATACTCGT	TCAAAGCTCTCCATTGACCTCGCCA
ipt3-2	12123	ACCCTTCCCGCCGGAAATACTCGT	ATTGACCTCGCCACCTGTTTCCCC
ipt4-1	51673	GGTCAAATTCCGGCACACCAATCG	AGTCTCTGTTTCGCCACCACCGGA
nlp1-2	80714	AGTGTTTCTGTGGATGAGCAACGC	GCACCTACATGTAGTCCTGGACTT
nlp4-1	106236	CCAACAGAGGAACCTGGATTTGCCA	TGTGAAGCCATGGAGCATTGAAGC

Supplementary table 3: recipe of 1/4 Long Ashton

For 1 L 1/4 Long Ashton

Macroelements	Final concentration
KH2PO4	0.1875 mM
MgSO4・7 H2O	0.1875 mM
Fe-EDTA	0.025 mM
CaCl2・2H2O	0.15 M

Microelements

MnCL2・4H20	2.5 uM
H3BO3	12.5 uM
ZnCl2	0.4375 uM
CuCl2	0.125 uM
Na2MoO4	0.2 uM
KI	0.25 uM
CoCl2・6H2O	0.025 uM

use 1M PIPES to adjust PH to 6.8

Supplementary table 4: gene expression in RNA-seq (fold change Log2)

symbiotic genes

ID	Gene	N_15min	N_30min	N_1hr	N_24h	N_3day
LotjaGi1g1v0062600	NFR1	0.233452	-0.13409	0.025939	-1.21213	-0.09474
LotjaGi2g1v0394950	NFR5	1.738167	1.450396	1.918582	-1.02996	0.990537
LotjaGi2g1v0330500	SYMRK	0.551038	0.278532	0.515252	-0.47826	0.417963
LotjaGi2g1v0352700	HMGR1	0.435194	-0.00379	-0.71145	-1.26666	-0.40824
LotjaGi1g1v0175100	NUP85	-0.2453	0.065377	-0.1897	0.78871	0.298218
LotjaGi1g1v0009600	NUP133	0.156583	0.135258	-0.04421	0.459827	0.572492
LotjaGi1g1v0800800	CASTOR	-0.21608	0.024791	0.089599	-0.24536	0.073006
LotjaGi6g1v0363700	POLLUX	-0.21816	-0.20502	-0.03447	-0.01654	0.015477
LotjaGi3g1v0307700	CCaMK	0.407834	0.20328	0.262606	-0.68605	0.172095
LotjaGi2g1v0343300	CYCLOPS	0.442067	0.073709	0.299389	-0.54264	-0.34678
LotjaGi3g1v0414350	NSP1	1.757873	2.247975	2.653475	0.875206	1.059292
LotjaGi1g1v0257100	NSP2	0.339974	-0.25001	-0.0133	-2.01805	-1.46349
LotjaGi1g1v0001500	NIN	0.28485	-0.92396	-1.42321	-2.82617	-1.92748
LotjaGi1g1v0643700	ERN1	1.200343	0.405313	0.138719	-0.68786	0.17782

nitrate marker genes

ID	Gene	N_15min	N_30min	N_1hr	N_24h	N_3day
LotjaGi3g1v0487600	*Nrt2.1a*	1.918003	1.997063	2.454819	1.954898	4.841707
LotjaGi3g1v0487700_L	*Nrt2.1b*	2.989983	3.508318	3.782086	1.773012	2.414457
LotjaGi1g1v0152000	*Nia*	2.965	4.731996	5.539115	4.878715	5.572681
LotjaGi4g1v0074400	*Nir*	3.249313	4.296373	5.122131	4.033633	5.269419
LotjaGi2g1v0280600	*Nigt1*	1.981943	2.765247	3.199917	2.637179	3.561792
LotjaGi4g1v0106500	*Cep1*	-0.0211	-0.22181	-1.08941	-2.93361	-4.00101
LotjaGi4g1v0241000	*Cep7*	0.179133	0.095163	-0.42652	-3.38989	-5.35008

cytokinin biosynthesis genes

ID	Gene	N_15min	N_30min	N_1hr	N_24h	N_3day
LotjaGi5g1v0339400	IPT1	-0.18038	-0.15925	0.275436	0.955232	-0.00332
LotjaGi4g1v0351300	IPT2	-0.55423	-0.23139	-0.17794	-0.03395	-0.90799
LotjaGi5g1v0164300	IPT3	-0.76763	-0.27093	-0.17163	-0.16516	-0.29385
LotjaGi6g1v0298900	IPT4	-0.36776	0.238909	0.671592	-0.04559	-1.6264
LotjaGi3g1v0480000	IPT5	0.20205	0.189835	0.355403	0.084323	-0.11047
LotjaGi3g1v0156900	IPT9	-0.1721	0.068239	0.032092	0.381893	0.239838
LotjaGi1g1v0735700	LOG1	-0.15874	-0.31733	-0.64082	-0.40141	-0.31564
LotjaGi1g1v0690800	LOG2	-0.19832	-0.0253	0.678125	1.728978	1.229952
LotjaGi5g1v0102900	LOG3	0.124695	-0.0489	-0.03451	-0.69139	0.033429
LotjaGi1g1v0427900	LOG4	0.688711	-0.20945	-0.15546	-0.67002	0.098455
LotjaGi3g1v0164600	LOG5	0.988409	-0.06328	0.788053	1.11271	0.84424
LotjaGi1g1v0368200	LOG6	0.223884	-0.14326	0.143556	-0.09636	-0.06494
LotjaGi2g1v0358500	LOG7	0.188094	0.542742	0.793825	1.00734	0.090812
LotjaGi3g1v0551100	LOG8	0.266459	0.143933	0.735254	-1.08074	-1.28175
LotjaGi5g1v0079100	LOG9	-0.59015	-0.03116	0.3134	0.499521	1.326805
LotjaGi6g1v0150400	LOG10	0.019454	0.002762	-0.38199	-0.11977	0.520784
