# Supplementary material for: Nitrate restricts nodule organogenesis through inhibition of cytokinin biosynthesis in Lotus japonicus
Source: Nat Commun. 2021 Nov 11;12:6544. doi: 10.1038/s41467-021-26820-9 (PMC8585978; doi:10.1038/s41467-021-26820-9)
Supplement: Supplementary file 3 — Description of Additional Supplementary Files [file 41467_2021_26820_MOESM3_ESM.pdf]

Description of Additional Supplementary Files

File name: Supplementary Data 1

Description: Normalised read counts for all genes

File name: Supplementary Data 2

Description: RNAseq differential expression statistics
