# Supplementary material for: Nitrate restricts nodule organogenesis through inhibition of cytokinin biosynthesis in Lotus japonicus
Source: Nat Commun. 2021 Nov 11;12:6544. doi: 10.1038/s41467-021-26820-9 (PMC8585978; doi:10.1038/s41467-021-26820-9)
Supplement: Supplementary file 6 — Reporting Summary [file 41467_2021_26820_MOESM6_ESM.pdf]

nature portfolio

Corresponding author(s): Dugald Reid

Last updated by author(s): Oct 13, 2021

Reporting Summary

Nature Portfolio wishes to improve the reproducibility of the work that we publish. This form provides structure for consistency and transparency in reporting. For further information on Nature Portfolio policies, see our Editorial Policies and the Editorial Policy Checklist.

Statistics

For all statistical analyses, confirm that the following items are present in the figure legend, table legend, main text, or Methods section.

n/a	Confirmed	
☐	☒	The exact sample size (*n*) for each experimental group/condition, given as a discrete number and unit of measurement
☐	☒	A statement on whether measurements were taken from distinct samples or whether the same sample was measured repeatedly
☐	☒	The statistical test(s) used AND whether they are one- or two-sided<br>*Only common tests should be described solely by name; describe more complex techniques in the Methods section.*
☐	☒	A description of all covariates tested
☐	☒	A description of any assumptions or corrections, such as tests of normality and adjustment for multiple comparisons
☐	☒	A full description of the statistical parameters including central tendency (e.g. means) or other basic estimates (e.g. regression coefficient) AND variation (e.g. standard deviation) or associated estimates of uncertainty (e.g. confidence intervals)
☐	☒	For null hypothesis testing, the test statistic (e.g. *F*, *t*, *r*) with confidence intervals, effect sizes, degrees of freedom and *P* value noted<br>*Give P values as exact values whenever suitable.*
☒	☐	For Bayesian analysis, information on the choice of priors and Markov chain Monte Carlo settings
☒	☐	For hierarchical and complex designs, identification of the appropriate level for tests and full reporting of outcomes
☒	☐	Estimates of effect sizes (e.g. Cohen's *d*, Pearson's *r*), indicating how they were calculated

Our web collection on statistics for biologists contains articles on many of the points above.

Software and code

Policy information about availability of computer code

Data collection	Data is stored in excel and raw data provided as a supplemental file. Processing of data for RNAseq and qPCR is described in the manuscript methods
Data analysis	R v4.1 was used for all data analysis, Waters IntelliStart MS Console was used for acquiring MS data, Salmon version 0.14.1 was used for RNAseq mapping, DeSeq2 (available from bioconductor) was used for RNAseq analysis

For manuscripts utilizing custom algorithms or software that are central to the research but not yet described in published literature, software must be made available to editors and reviewers. We strongly encourage code deposition in a community repository (e.g. GitHub). See the Nature Portfolio guidelines for submitting code & software for further information.

Data

Policy information about availability of data

All manuscripts must include a data availability statement. This statement should provide the following information, where applicable:

- Accession codes, unique identifiers, or web links for publicly available datasets
- A description of any restrictions on data availability
- For clinical datasets or third party data, please ensure that the statement adheres to our policy

RNAseq raw data has been submitted to NCBI under BioProject accession number PRJNA642098. Cytokinin measurements have been deposited at Metabolights under submission number MTBLS3492. All raw data used for producing figures is available in the source data file.

Field-specific reporting

Please select the one below that is the best fit for your research. If you are not sure, read the appropriate sections before making your selection.

- [x] Life sciences
- [ ] Behavioural & social sciences
- [ ] Ecological, evolutionary & environmental sciences

For a reference copy of the document with all sections, see nature.com/documents/nr-reporting-summary-flat.pdf

Life sciences study design

All studies must disclose on these points even when the disclosure is negative.

Sample size	Sample size was not predetermined. Sample sizes were in line with norms of the field and determined by previous experience in performing experiments of the same technical nature
Data exclusions	No data was excluded from the manuscript
Replication	Biological replicates were used in all experiments. Technical replication was performed in qPCR which was averaged before comparing biological replicates
Randomization	All research material was homozygous, and therefore individual organisms were randomly distributed to control and treatment groups
Blinding	Experimenters were not blind to sample collection, but tubes were assigned numbers which were reconciled with sample names once molecular analysis was completed

Reporting for specific materials, systems and methods

We require information from authors about some types of materials, experimental systems and methods used in many studies. Here, indicate whether each material, system or method listed is relevant to your study. If you are not sure if a list item applies to your research, read the appropriate section before selecting a response.

Materials & experimental systems

n/a	Involved in the study	
x		Antibodies
x		Eukaryotic cell lines
x		Palaeontology and archaeology
x		Animals and other organisms
x		Human research participants
x		Clinical data
x		Dual use research of concern

Methods

n/a	Involved in the study	
x		ChIP-seq
x		Flow cytometry
x		MRI-based neuroimaging
